# Absence of Nosocomial Transmission of Imported Lassa Fever during Use of Standard Barrier Nursing Methods

**DOI:** 10.3201/eid2406.172097

**Published:** 2018-06

**Authors:** Anna Grahn, Andreas Bråve, Thomas Tolfvenstam, Marie Studahl

**Affiliations:** University of Gothenburg, Gothenburg, Sweden (A. Grahn, M. Studahl);; Public Health Agency of Sweden, Solna, Sweden (A. Bråve);; Karolinska University Hospital, Stockholm, Sweden (T. Tolfvenstam)

**Keywords:** nosocomial transmission, imported Lassa fever, Lassa fever, hemorrhagic fevers, Lassa virus, LASV, viruses, contacts, standard barrier nursing methods, disease transmission, infectious diseases, zoonoses, Sweden

## Abstract

Nosocomial transmission of Lassa virus (LASV) is reported to be low when care for the index patient includes proper barrier nursing methods. We investigated whether asymptomatic LASV infection occurred in healthcare workers who used standard barrier nursing methods during the first 15 days of caring for a patient with Lassa fever in Sweden. Of 76 persons who were defined as having been potentially exposed to LASV, 53 provided blood samples for detection of LASV IgG. These persons also responded to a detailed questionnaire to evaluate exposure to different body fluids from the index patient. LASV-specific IgG was not detected in any of the 53 persons. Five of 53 persons had not been using proper barrier nursing methods. Our results strengthen the argument for a low risk of secondary transmission of LASV in humans when standard barrier nursing methods are used and the patient has only mild symptoms.

Lassa fever is a potentially severe viral hemorrhagic illness caused by Lassa virus (LASV). The reported mortality rate is 1% overall but can be up to 15%–20% for hospitalized patients (*1*,*2*). LASV is normally transmitted to humans by ingested or inhaled rodent (*Mastomyces natalensis*) excreta, mainly urine. However, person-to-person transmission can occur by contact with infected body fluids. Treatment with ribavirin has been shown to reduce mortality rates when administered early during infection (*3*) and has been used for postexposure prophylaxis.

The types of personal protective equipment (PPE) and medical facility to use when caring for Lassa fever patients have been discussed (*4*–*6*). In disease-endemic areas, such as Liberia, hospital staff has been reported to have higher prevalence of antibodies against LASV than the general village population (*7*,*8*). However, a prospective study conducted in Sierra Leone (*6*) showed no increased risk for nosocomial transmission when standard barrier nursing methods, including gloves, gowns, and masks with various rates of compliance, were used. In countries to which LASV is not endemic, risk for nosocomial transmission has been reported to be low in persons caring for the hospitalized index patient, even without more special precautions than barrier nursing methods (*9*–*12*). Serologic studies in these countries have not demonstrated nosocomial transmission resulting in infections in healthcare workers (*9*,*10*,*13*).

The recommendations of the US Centers for Disease Control and Prevention are to use barrier nursing methods, including gloves, gowns, masks, and goggles, and an isolation room when caring for a patient with Lassa fever (*14*). The World Health Organization (WHO) recommends gloves, long-sleeved gowns, and face shields or masks and goggles when caring for the patient and being within 1 m of the patient (*15*). In countries to which Lassa fever is not endemic, these patients are cared for mostly in high-level isolation units, at least after a diagnosis is made. However, these units are expensive, labor-intensive, and strenuous for the patient. In addition, the number of patients that can be treated in high-isolation facilities simultaneously is often limited within a country. We investigated whether LASV infection occurred in healthcare workers who used standard barrier nursing methods during the first 15 days of caring for a patient with Lassa fever in Sweden.

## Materials and Methods

### Index Patient

A 72-year-old woman was admitted to Sahlgrenska University Hospital in Gothenburg, Sweden, in March 2016, ten days after onset of fever, nausea, arthralgia, loose stools, and headache, and 2 days after onset of personality changes (*16*). The initial fever had resolved after 3 days and 7 days before hospitalization. The patient and her husband had visited Liberia for 6 weeks and returned to Sweden 5 days before onset of primary symptoms. After more common diagnoses had been ruled out and a hearing deficit developed in the patient, Lassa fever was suspected. Fourteen days after admission, the patient was given a diagnosis of Lassa fever after detection of LASV IgG and low titers of LASV IgM; LASV RNA was also detectable by PCR.

The patient had loose stools and vomited on 2 occasions during the first 14 days of hospitalization but had no fever (temperature >38°C). She needed help with hygiene issues, including toilet visits. Fifteen days after admission, the day when the diagnosis was confirmed by PCR, she was transferred to a high-level isolation unit. We detected LASV RNA by using PCR in samples from serum, whole blood, urine, and feces obtained during the first 15 days of hospitalization. The highest concentration of LASV RNA detected was 1.2 × 10^5^ copies/mL. For other characteristics of this patient, see the Figure and Grahn et al. (*16*).

**Figure:**
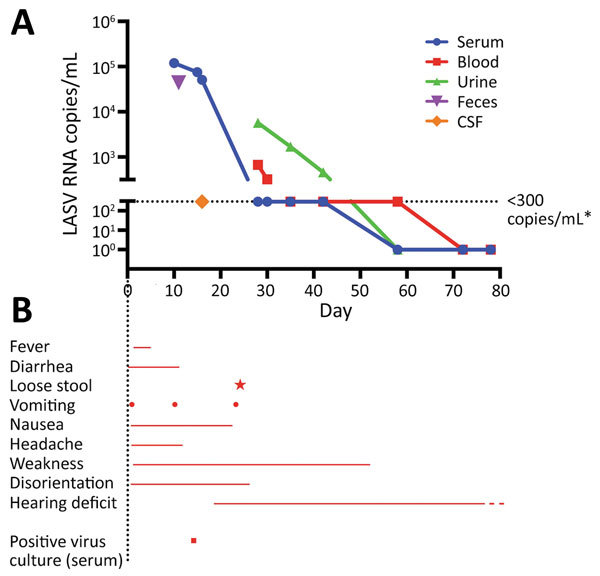
Characteristics of index patient (72-year-old woman) with imported Lassa fever, Gothenburg, Sweden. A) Results of virus PCR. B) Signs and symptoms and positive serum culture result. Symbols indicate days when signs/symptoms occurred and day of positive serum culture result, and lines indicate continuing signs/symptoms. Dashed red line indicates a decrease in this symptom. *The limit of quantitation of LASV was 300 copies/mL, and detectable but not quantifiable levels of LASV were defined as <300 copies/mL. Day 10 is the day of hospital admission. CSF, cerebrospinal fluid; LASV, Lassa virus.

### Contacts

After making a diagnosis for the index patient, 15 days after admission, risk assessment and management of contacts were performed as reported (Table 1) (*11*). A high risk was defined as having unprotected exposure of damaged skin or mucous membranes (e.g., mucosal exposure to splashes, needlestick injury) to potentially infectious blood or body fluids, or unprotected handling of clinical/laboratory specimens. A low risk was defined as having close direct contact with the patient (e.g., routine medical/nursing care, handling of clinical/laboratory specimens) and using barrier nursing methods when handling body fluids. Barrier nursing methods used before diagnosis were basic hygiene procedures and PPE, including gloves and plastic apron without sleeves when at risk for direct contact with body fluids from the patient. No face masks were used. After diagnosis (15 days after admission), facial shield (without face mask) was added to the PPE.

**Table 1:** Level of risk related to exposure to a patient with Lassa fever and action, by category*

Risk category	Description	Action
No risk (category 1)	No contact with the case-patient; casual contact with the case-patient (e.g., sharing room without direct contact with any potentially infectious material)	Inform of absence of risk; give category 1 (general) fact sheet
Low risk (category 2)	Close direct contact with the case-patient (e.g., general routine medical/nursing care, handling of clinical/laboratory specimens), but did not handle body fluids or wore personal protective equipment appropriately	Self monitor† for fever and other symptoms compatible with Lassa fever; report to the safety officer nurse if fever >38°C or new somatic symptoms, with further evaluation as necessary; give category 2 fact sheet
High risk† (category 3)	Unprotected exposure of damaged skin or mucous membranes (e.g., mucosal exposure to splashes, needlestick injury) to potentially infectious blood or body fluids; or unprotected handling of clinical laboratory specimens	Report own temperature daily‡ and report this temperature and any new somatic symptoms to the safety officer nurse every day, with further evaluation as necessary; give category 3 fact sheet

*Adapted from Kitching et al. (*11*). †Consideration for ribavirin prophylaxis within this group. ‡Contacts to be monitored for 21 d from last possible exposure to the case-patient.

Contacts at possible risk (low or high risk) were monitored 21 days postexposure. This monitoring included measuring body temperature twice a day and awareness of any new symptoms. If fever or any symptoms appeared, these contacts were informed to contact an established safety officer. A total of 80 contacts were identified as at possible risk. Seventy-six contacts were personnel or students at the Sahlgrenska University Hospital who had close contact with the index patient or handled her body fluids. Four contacts were family members. All 80 contacts were identified as at possible risk and were categorized into low-risk exposures at the time when the diagnosis of the index patient was made. In addition, 45 personnel at the high-level isolation unit, all of them having used enhanced protective equipment (including powered air purifying respirator) when caring for the patient, were also monitored as a safety routine.

As part of this study, all 76 healthcare workers at possible risk were again assessed through interviews conducted by 2 study doctors according to a more detailed questionnaire. Questions were asked to evaluate the contact with the index patient or her body fluids and timing of the contacts. Contacts were asked to participate in the study and provide serum samples after >2 incubation periods (2 × 21 days).

This study was approved by the Medical Ethics Committee at Gothenburg University, and written informed consent was obtained for inclusion in the study. The study was performed in accordance with ethics standards in the Declaration of Helsinki and its later amendments.

### Analyses for LASV IgG

We stored serum samples at –70°C. We performed serologic analysis by using an immunofluorescence test with LASV strain SL-NL (002v EVA880)–infected confluent Vero cells. Serum samples were analyzed for LASV-specific IgG in 2-fold serial dilutions starting at 1:10. Serum from index the patient obtained 3 months after onset of disease was used as positive control (IgG titer >1:2,560).

## Results

Of the 76 personnel who were defined as being at possible risk, we included 53 in the study. The remaining 23 personnel were not included because we were unable to contact them despite several telephone calls or because they were unable to report for blood sampling. We obtained demographic characteristics for the 53 personnel (Table 2). The included personnel provided blood samples for a median of 77 days (range 69–110 days), and samples were analyzed for LASV IgG. Antibodies were not detected in any samples from the 53 personnel.

**Table 2:** Characteristics of 53 of 76 persons at Sahlgrenska University Hospital, Gothenburg, Sweden, who had contact with case-patient with Lassa fever*

Contact classification	Total	Sex, F:M	Mean age, y (range)	Low risk	High risk
Doctor	3	1:2	38 (28–49)	3	0
Nursing/AHP	28	26:3	35 (22–58)	27	2
Laboratory staff	18	17:1	45 (22–62)	18	0
Radiology	2	2:0	NA	2	0
Medical students	1	0:1	NA	1	0
Total	53	46:7	39 (22–62)	51	2

*AHP, allied health professionals (e.g., physiotherapist and occupational therapist); NA, not analyzed because of confidentiality reasons.

Of the 53 personnel in the study, 15 had different symptoms of illness during the incubation period (influenza-like symptoms, including sore throat, cough, and rhinitis, n = 12; stomachache, nausea, or vomiting, n = 3; and symptoms of urinary infection, n = 1). Five of these 15 personnel had a temperature >38°C. The 5 personnel who reported fever in conjunction with symptoms possibly suggestive of Lassa fever also provided blood samples during the incubation period. Test results for LASV RNA were negative.

Twelve of the 53 personnel had obtained blood (n = 11), cerebrospinal fluid (n = 1), urine (n = 1), or feces (n = 1) samples from the index patient. Twenty personnel had assisted the patient during lavatory visits and dealt with stool, urine, or both from the patient as a result. Seventeen laboratory personnel had handled blood, cerebrospinal, stool, and urine samples from the patient.

Five personnel and 1 medical student reported that they were not wearing gloves when in close contact with or handling specimens from the patient. Of these 6 persons, 2 doctors, 1 physiotherapist, 1 occupational therapist, and 1 medical student performed physical examinations, and 1 of the laboratory staff did not wear gloves when handling agar plates containing blood from the patient. Two of these 6 persons reported influenza-like symptoms, and 1 of these 2 persons reported a temperature >38°C during the incubation period.

In addition, 5 personnel who were wearing gloves and plastic aprons were exposed to body fluids from the index patient on unprotected undamaged skin or mucous membranes. Of these 5 personnel, 1 was possibly exposed to profuse vomitus on mucous membranes, 1 was possibly exposed to droplets of saliva on her face after the index patient coughed, and 3 were exposed to urine on undamaged unprotected skin. Four of these 5 personnel reported influenza-like symptoms (n = 3) or stomach ache (n = 1); 3 of the 4 reported a temperature >38°C during the incubation period.

Of the 53 personnel, 2 were categorized as being at high risk; all others were categorized as being at low risk after interviews had been conducted. These findings were in contrast to categorization during the acute phase, when all personnel were categorized as being at low risk. During the acute phase, information regarding high-risk exposures was misjudged or inappropriate probably because of less structured questions during the acute phase and a stressful situation. The 2 nurses who had been categorized as being at high risk after the study interview was conducted were the 1 possibly exposed to profuse vomitus on mucous membranes and the 1 possibly exposed to droplets of saliva on her face after the index patient coughed.

## Discussion

The lack of LASV IgG in blood samples from all included personnel who had been exposed to the index patient supports the suggestion that risk is probably low for hospital transmission of LASV when standard barrier nursing methods are used, at least when the symptoms are mild with few occasions of vomiting and diarrhea of the index patient. These results are consistent with those of previous studies, which showed a low risk for person-to-person transmission in hospital settings (*6*,*9*–*11*,*13*,*17*). In countries to which LASV is not endemic, ≈40 cases of imported Lassa fever have been reported since LASV was first identified in 1969, and only 1 case of secondary transmission in this type of country has been reported (*18*). The secondary case was a funeral home employee who had been handling a body before a diagnosis of Lassa fever was made at postmortem analyses.

In addition, 1 case of probable nosocomial transmission was reported in Germany in 2000 (*5*). A physician who had been examining a patient with Lassa fever and not using any barrier nursing methods was found to be reactive for LASV IgG when high-risk and medium-risk contacts were screened for LASV IgG. These antibodies were specific for the LASV strain isolated from the index patient. However, no specific increase in antibody titer was observed, and secondary transmission could not be confirmed. In the index patient in this study, virus RNA concentration in serum increased concomitant with disease progression. Other than the report from Germany (*5*), 3 studies have been conducted in countries to which LASV is not endemic in which serologic testing showed no asymptomatic infections in contact persons, including medium-risk and high-risk contacts (*9*,*10*,*13*). Our study strengthens the argument that the risk for asymptomatic infections in nosocomial settings is modest, at least if the disease manifests with relatively mild symptoms.

In our study, the index patient was provided care for 15 days with standard barrier nursing methods. A delay in diagnosis is not uncommon for imported cases (*4*,*11*,*19*) and is probably at least partly dependent on often nonspecific symptoms for Lassa fever. During these 15 days, 5 personnel and 1 medical student were in close contact with the index patient or with specimens from the patient, without use of barrier methods. In addition, 5 other personnel were exposed to body fluids from the index patient on unprotected skin, 1 of them possibly on mucous membranes. Indeed, risk for nosocomial transmission can be even higher before diagnosis when use of barrier nursing methods might be inappropriate, as in the case in Germany (*5*). Other situations with high risk for nosocomial transmission include profuse excretion of body fluids, such as vomitus, watery stool, or blood with high amounts of virus shedding, or invasive care in an intensive care unit. To avoid nosocomial transmission, barrier nursing methods must always be used by healthcare workers when caring for patients with potentially contagious diseases, according to national recommendations.

Moreover, it is not evident how to categorize the contacts, and there are different suggestions in the literature (*11*,*18*,*20*,*21*). It is also useful to ask structured questions and to follow criteria strictly when categorizing contacts to avoid incorrect categorization, as was seen in our study. In addition, it is not evident that all viral hemorrhagic fevers should be categorized the same way because there are considerable differences in illness and mortality rates for various viral hemorrhagic fevers (e.g., between Lassa fever and Ebola). In our study, all contacts except for 2 were categorized as being at low risk despite exposure of unprotected skin to body fluids or close physical contact with the index patient. This type of categorization is in contrast to that of the study in Germany, in which these types of contacts were categorized as high risk and prophylaxis with oral ribavirin was administered (*5*). However, ribavirin is associated with side effects, including pancreatitis and liver injury (*22*). In addition, prophylactic efficacy has not been demonstrated for humans. Because no secondary transmission of LASV has been proven in contacts with exposure of body fluids to unprotected skin or close physical contact, including in our study, we regard the categorization of Kitching et al. (*11*) as reasonable. The criteria for high-risk contacts in that study included exposure of body fluids to mucous membranes or damaged skin, such as by needle injury. Of the exposed personnel in our study, no one received ribavirin after risk classification and categorization. Thus, we agree with Kitching et al. that prophylaxis with ribavirin should only be considered in the instance of confirmed, extensive exposure to potentially infected body fluids.

Another aspect of transmission is the degree of illness. A patient with severe symptoms, including profuse vomiting, diarrhea, and bleeding, implies a high risk for virus transmission, and one could also presume that the degree of illness reflects the viremia (*23*), although data on the association between degree of illness and amount of infectious virus in different body fluids are scarce (*5*,*16*,*24*). However, despite the absence of fever 7 days before admission, the index patient in the present study had moderately high concentrations of LASV RNA in serum at admission and traces of viral RNA (<300 copies/mL) up to 32 days after admission. In addition, she had detectable virus RNA in feces and urine, and virus might be shed from the urine for a long time after recovery (*25*).

Whether detectable LASV RNA in different body fluids represents living virus or only the viral genome incorporated into dead and dying cells from necrotic tissue that might gain direct access to the circulation is unknown. It is also not clear how transmissible LASV is at different concentrations in different body fluids. Only virus cultivation can determine whether body fluids contain replication-competent virus. In a recent study, results for LASV cultivation in blood were positive for up to 11 days but were negative after fever resolution, although LASV RNA was detectable for a longer period in different body fluids (*24*). In our study, results of virus cultivation in serum were positive for up to 16 days, even after fever resolution. However, cultivation is difficult to perform, especially from materials such as urine or feces, and is not sufficiently sensitive. Thus, there is no useful method available for distinguishing virus RNA/DNA in different body fluids from living virus or dead and dying cells from necrotic tissue, a prerequisite for evaluating the extent of contagiousness.

Last, we have verified that barrier nursing methods are not consistently defined. In our study, we used basic hygiene procedures, including use of gloves and plastic aprons when persons were at risk for direct contact with body fluids, before diagnosis. Definition of barrier nursing methods of WHO and the Centers for Disease Control and Prevention when caring for a patient with suspected or confirmed Lassa fever includes gloves, gowns, and facial shields or masks and goggles, although WHO emphasizes that these precautions are most necessary when being in close contact (<1 m) with the patient.

In summary, our study strengthens the argument for low risk of secondary transmission of LASV in humans when proper basic nursing methods are used and the disease manifests with relatively mild symptoms. The adequate safety level when caring for patients with suspected or confirmed Lassa fever in countries to which LASV is not endemic should be discussed. Further studies of how infectivity varies depending on severity of symptoms and route of transmission are essential.
